# Organelle Optogenetics: Direct Manipulation of Intracellular Ca^2+^ Dynamics by Light

**DOI:** 10.3389/fnins.2018.00561

**Published:** 2018-08-17

**Authors:** Toshifumi Asano, Hiroyuki Igarashi, Toru Ishizuka, Hiromu Yawo

**Affiliations:** ^1^Department of Cell Biology, Graduate School of Medical and Dental Sciences, Tokyo Medical and Dental University, Tokyo, Japan; ^2^Department of Physiology and Pharmacology, Tohoku University Graduate School of Medicine, Sendai, Japan; ^3^Department of Developmental Biology and Neuroscience, Tohoku University Graduate School of Life Sciences, Sendai, Japan

**Keywords:** channelrhodopsin, endoplasmic reticulum, sarcoplasmic reticulum, ER/SR, muscle, C2C12, store-operated Ca^2+^ entry (SOCE), super-resolution microscopy

## Abstract

As one of the ubiquitous second messengers, the intracellular Ca^2+^, has been revealed to be a pivotal regulator of various cellular functions. Two major sources are involved in the initiation of Ca^2+^-dependent signals: influx from the extracellular space and release from the intracellular Ca^2+^ stores such as the endoplasmic/sarcoplasmic reticulum (ER/SR). To manipulate the Ca^2+^ release from the stores under high spatiotemporal precision, we established a new method termed “organelle optogenetics.” That is, one of the light-sensitive cation channels (channelrhodopsin-green receiver, ChRGR), which is Ca^2+^-permeable, was specifically targeted to the ER/SR. The expression specificity as well as the functional operation of the ER/SR-targeted ChRGR (ChRGR_ER_) was evaluated using mouse skeletal myoblasts (C2C12): (1) the ChRGR_ER_ co-localized with the ER-marker KDEL; (2) no membrane current was generated by light under whole-cell clamp of cells expressing ChRGR_ER_; (3) an increase of fluorometric Ca^2+^ was evoked by the optical stimulation (OS) in the cells expressing ChRGR_ER_ in a manner independent on the extracellular Ca^2+^ concentration ([Ca^2+^]_o_); (4) the Δ*F*/*F*_0_ was sensitive to the inhibitor of sarco/endoplasmic reticulum Ca^2+^-ATPase (SERCA) and (5) the store-operated Ca^2+^ entry (SOCE) was induced by the OS in the ChRGR_ER_-expressing cells. Our organelle optogenetics effectively manipulated the ER/SR to release Ca^2+^ from intracellular stores. The use of organelle optogenetics would reveal the neuroscientific significance of intracellular Ca^2+^ dynamics under spatiotemporal precision.

## Introduction

The intracellular Ca^2+^, as one of the second messengers, plays a pivotal role in any kind of cell by conducting information (Berridge et al., 1998; Bagur and Hajnóczky, 2017). The Ca^2+^ signals that emerge from the external input, such as extracellular signaling molecules, are subsequently transduced for the activation of various signaling molecules to coordinate a wide variety of cell functions (Kakiuchi and Yamazaki, 1970; Berridge et al., 2003). When a murine myoblast, C2C12, was compelled to express one of the chimeric channelrhodopsins, channelrhodopsin-green receiver (ChRGR), a patterned optical stimulation (OS) induced an oscillation of membrane potential and accelerated the assembly of sarcomere, the smallest contractile unit in muscle fibers in a manner dependent on the increase of intracellular Ca^2+^ ([Ca^2+^]_i_) (Asano et al., 2015). Therefore, the cyclic [Ca^2+^]_i_ elevation is assumed to be necessary for the sarcomere assembly. However, it has yet to be elucidated whether it is a sufficient condition without Ca^2+^ influx through a plasma membrane.

So far, the mobilization of intracellular Ca^2+^ has been investigated through pharmacological methods using drugs or caged compounds (Morad et al., 1988; Kaplan and Somlyo, 1989; Adams et al., 1997). However, these methods are limited in the spatiotemporal resolution because of the rapid diffusion of reagents in the cytoplasm. On the other hand, the optical control of Ca^2+^ signaling in mammalian cells would render two major advantages over conventional approaches: high spatiotemporal resolution and tunability of the magnitude in a manner dependent on the light energy. The Ca^2+^ signaling could be initiated through either influx from the extracellular space or efflux from the internal Ca^2+^ stores such as the endoplasmic/sarcoplasmic reticulum (ER/SR) (Bagur and Hajnóczky, 2017). Here, we established our state-of-the-art method “organelle optogenetics.” This method manipulates the Ca^2+^ release from Ca^2+^ stores under high spatiotemporal precision. In fact, one of the light-responsive cation channels (ChRGR) was specifically targeted to ER/SR, and subsequently illuminated to induce Ca^2+^ release from these organelles as well as their depletion.

## Materials and methods

### Plasmids

A cDNA fragment encoding ChRGR-Venus (Wen et al., 2010) was amplified by PCR and subcloned into EcoRI sites in pCAGGS by In-Fusion cloning (Takara Bio, Shiga, Japan). To generate a ChRGR with an ER-retention motif (ChRGR_ER_), a cDNA encoding Gln^4765^-Ile^4866^ of mouse ryanodine receptor 2 (NM_023868) was inserted in-frame between ChRGR and Venus using the AgeI restriction site by In-Fusion cloning. The construct was verified by DNA sequencing.

### Cell culture and transfection

The current-voltage (*I-V*) relationship of the ChRGR photocurrent was assessed using the ND 7/23 cell—a hybrid cell lines derived from neonatal rat dorsal root ganglia neurons fused with the mouse neuroblastoma (Wood et al., 1990). ND 7/23 cells were grown on a poly-L-lysine (Sigma-Aldrich, St Louis, MO)-coated coverslip in Dulbecco's modified Eagle's medium (DMEM, Wako Pure Chemical Industries, Osaka, Japan) supplemented with 10% fetal bovine serum (Biological Industries, Kibbutz Beit-Haemek, Israel) under a 5% CO_2_ atmosphere at 37°C. The cells were maintained for no more than ten passages and grown to 80–90% confluence in the culture dish. The expression plasmids were transiently transfected in ND 7/23 cells using Effectene Transfection Reagent (Qiagen, Hilden, Germany) according to the manufacturer's instructions. The medium was supplemented with 2.5 μM all-*trans* retinal at 6 h after transfection. Electrophysiological recordings were then conducted 24-48 h after the transfection. Successfully transfected cells were identified by the presence of Venus fluorescence.

The C2C12 cell is a myoblast line derived from mouse skeletal muscle (RIKEN Cell Bank, Tsukuba, Japan), which has been used as one of model systems of skeletal muscle development and differentiation. The cells were maintained for no more than fifteen passages and kept at 37°C with a 5% CO_2_ atmosphere in Dulbecco's Modified Eagle's Medium (DMEM, Wako Pure Chemical Industries), which was supplemented with 20% fetal bovine serum (Invitrogen, Carlsbad, CA), 100 units/mL penicillin, and 100 μg/mL streptomycin (Sigma-Aldrich). C2C12 myoblasts were grown to 80–90% confluence on a collagen-coated coverslip, transfected with plasmids using either Effectene or Lipofectamine 2000 (Invitrogen) and used for patch clamp and calcium imaging experiments.

### Electrophysiology

All experiments were conducted at room temperature (23 ± 2°C). The *I-V* relationship of the ChRGR photocurrent was investigated as previously described (Ishizuka et al., 2006) using an EPC-8 amplifier (HEKA Electronic, Lambrecht, Germany) under a whole-cell patch clamp configuration. The data were filtered at 1 kHz, sampled at 10 kHz (Digdata1440 A/D, Molecular Devices Co., Sunnyvale, CA) and stored in a computer (pCLAMP 10.3, Molecular Devices). The internal pipette solution for whole-cell voltage-clamp recordings from ND7/23 cells contained (in m*mol*): 120 N-methy-D-glucamine, 50 tetraethylammonium (TEA)-Cl, 10 EGTA, 50 HEPES, 5 MgCl_2_, 2.5 MgATP, adjusted to pH 8.4 with HCl. The low Ca^2+^ extracellular solution contained (in m*mol*): 100 N-methy-D-glucamine, 50 HEPES, 0.1 CaCl_2_, 46.9 MgCl_2_, 11 glucose, adjusted to pH 8.4 with HCl whereas the high Ca^2+^ solution contained (in m*mol*): 100 N-methy-D-glucamine, 50 HEPES, 10 CaCl_2_, 37 MgCl_2_, 11 glucose, adjusted to pH 8.4 with HCl. The directly measured liquid junction potential was −3.4 mV for either the low Ca^2+^ or the high Ca^2+^ external solution and was not compensated for. The photocurrent was evoked by a light from SpectraX light engine (Lumencor Inc., Beaverton, OR) at a wavelength (nm, >90% of the maximum) of 475 ± 28 nm. The power of light was directly measured under microscopy by a visible light-sensing thermopile (MIR-101Q, SSC Co., Ltd., Kuwana City, Japan) that was 1.3 mWmm^−2^ on the specimen.

The myoblasts expressing either ChRGR-Venus or that with the ER retention signal (ChRGR_ER_-Venus) were identified by Venus fluorescence using a conventional epifluorescence microscope (BX51WI, Olympus, Tokyo, Japan), which was equipped with a 60× water-immersion objective lens (LUMplanPl/IR60x, Olympus) and a filter cube (excitation, 495 nm; dichroic mirror, 505 nm; barrier filter, 515 nm). Photocurrents were recorded as previously described (Ishizuka et al., 2006) using an Axopatch 200B amplifier (Molecular Devices Co., Sunnyvale, CA) under a whole-cell patch clamp configuration. The data were filtered at 1 kHz, sampled at 10 kHz (Digdata1440A, Molecular Devices) and stored in a computer (pCLAMP 10.2, Molecular Devices). The standard extracellular Tyrode's solution contained (in m*mol*): 138 NaCl, 3 KCl, 2.5 CaCl_2_, 1.25 MgCl_2_, 10 HEPES, 4 NaOH, and 11 glucose (pH 7.4 adjusted with HCl). The standard patch pipette solution contained (in m*mol*): 120 CsOH, 100 glutamic acid, 0.2 EGTA, 10 HEPES, 2.5 MgCl_2_, 3 MgATP, 0.3 Na_2_GTP, and 0.1 leupeptin (pH 7.4 adjusted with CsOH) for the voltage clamp. To test the optical responses, we used a cyan LED (505 ± 15 nm, LXHL-NE98, Philips Lumileds Lighting Co., San Jose, USA), which was regulated by a pulse generator (SEN-7203, Nippon Koden, Japan) and pCLAMP 10.2 computer software. Its power density through an objective lens was 1.6 mWmm^−2^ when focused on the specimen.

### Immunohistochemistry

Cells were fixed with 4% paraformaldehyde/PBS and blocked in 0.1% Triton X-100/PBS containing 5% goat serum for 1 h. The samples were subsequently treated overnight with the primary antibodies, i.e., rat monoclonal anti-GFP IgG2a (1/1000, GF090R, Nacalai Tesque, Kyoto, Japan) and mouse monoclonal anti-KDEL (1/100, sc-58774, Santa Cruz Biotechnology, CA, USA) diluted in 0.1% Triton X-100/PBS containing 5% goat serum, and then incubated for 2 h with the secondary antibodies, i.e., Alexa Fluor 488-conjugated goat anti-rat IgG (Invitrogen) and Alexa Fluor 555-conjugated goat anti-mouse IgG (Invitrogen), diluted at 1/200 in 0.1% Triton X-100/PBS containing 5% goat serum. The specimens were washed three times with PBS between treatments and mounted with ProLong Gold antifade mounting medium (Invitrogen). These procedures were performed at room temperature. Super-resolution images were taken under TCS SP8 STED 3X (Leica, Wetzlar, Germany) with the STED (depletion) laser at 660 nm and white light laser. Alexa488 was excited at 488 nm and detected between 489 and 550 nm (gate, 1.5–6.5 ns) and Alexa555 was excited at 561 nm and detected between 563 and 680 nm (gate, 0.5–6.5 ns).

### Real time Ca^2+^ imaging

Cells were transfected with ChRGR_ER_ and the red fluorescent Ca^2+^ probe R-CaMP1.07 (Ohkura et al., 2012) plasmids simultaneously using Lipofectamine 2000. After 24 h, the red fluorescent signal of R-CaMP1.07 was acquired using a high-speed laser-scanning confocal microscopy system (A1R, Nikon, Tokyo, Japan) equipped with 16 × water-immersion objectives (0.8 NA), a 561-nm DPSS laser, a 405/488/561/640-nm dichroic mirror and a 580 ± 23 nm bandpass filter. The OS was given by high power 7-unit LED (450 ± 10 nm, LXML-PR01, Lumileds Lighting, USA). The images were sampled at 30 fps (resonant scan; 512 × 128 pixels) and analyzed using ImageJ software while regions of interest (ROIs) were each set to cover a single cell under visual identification. The fluorescence intensity in each ROI was sampled as the time series of digits and analyzed with Excel software (Microsoft Japan, Tokyo, Japan). The fluorescence change was defined as Δ*F*/*F*_0_ = (*F*_t_-*F*_0_)/*F*_0_, where *F*_t_ is the fluorescence intensity at time *t*, and *F*_0_ is the average baseline fluorescence 1 s before the stimulation. The averaged Δ*F*/*F*_0_ within 300 ms during the stimulation was used as the magnitude of the Ca^2+^ transient. The imaging experiment was performed under superfusion with normal extracellular Tyrode's solution containing (in m*mol*): 138 NaCl, 3 KCl, 2 CaCl_2_, 1 MgCl_2_, 10 HEPES, 4 NaOH, and 11 glucose (pH 7.4 adjusted with HCl), then switched to the Ca^2+^-free extracellular Tyrode's solution containing (in m*mol*): 138 NaCl, 3 KCl, 5 MgCl_2_, 10 HEPES, 4 NaOH, 1 Na_2_EGTA and 11 glucose (pH 7.4 adjusted with HCl).

The store depletion and the subsequent store-operated Ca^2+^ entry (SOCE) were investigated for the ChRGR_ER_-expressing C2C12 myoblasts co-transfected with R-GECO1, a red-shifted fluorescent Ca^2+^-sensor (Zhao et al., 2011), under superfusion with the Ca^2+^-free Tyrode's solution containing (in m*mol*): 138 NaCl, 3 KCl, 3.75 MgCl_2_, 10 HEPES, 4 NaOH, and 11 glucose (pH 7.4 adjusted with HCl), then switched to the standard Tyrode's solution containing (in m*mol*): 138 NaCl, 3 KCl, 2.5 CaCl_2_, 1.25 MgCl_2_, 10 HEPES, 4 NaOH and 11 glucose (pH 7.4 adjusted with HCl). In some experiments thapsigargin (TG, 2–5 μM, Tocris Bioscience, Bristol, UK) was included in the Ca^2+^-free solution. For the store depletion experiments, images of R-GECO1 fluorescence were acquired using a laser-scanning confocal microscopy system (A1R, Nikon) equipped with 16 × water-immersion objectives (0.8 NA). A fiber-coupled 451-nm laser source (Optohub, Saitama, Japan) was used for the optical stimulation. The free end of the optic fiber (core diameter; 50 μm, Doric Lenses, Quebec City, Canada) was placed close to the C2C12 cell. The power of light was directly measured at the free end of the optic fiber, and was 18.7 μW. For the SOCE experiments, images of R-GECO1 fluorescence were acquired every 10 s on a FV1200 confocal laser scanning microscope (Olympus) equipped with a 40 × UPlanSApo objective lens using the 559 nm excitation and 590 nm emission long pass filter sets. The OS (475 ± 10 nm, 2.8 mWmm^−2^) were applied at 20 Hz with 10 ms duration and 100 pulses between imaging sequences. The store-operated Ca^2+^ entry (SOCE) was monitored using Ca^2+^ add-back protocol after treating with either OS or TG in a Ca^2+^-free Tyrode's solution. The Δ*F/F*_0_ after Ca^2+^ add-back was expressed by averaging 20 s of peak value after changing [Ca^2+^]_o_.

### Statistics

All data in the text and figures are expressed as the mean ± SEM and evaluated using the Mann-Whitney *U*-test for the unpaired data, the Wilcoxon signed rank test for the paired data, and the one-way Kruskal-Wallis test by ranks for multi-group data to determine statistical significance, unless stated otherwise. It was judged as statistically insignificant when *P* > 0.05.

## Results

### Characterization of ER-targeting channelrhodopsin

**Figure 1:**
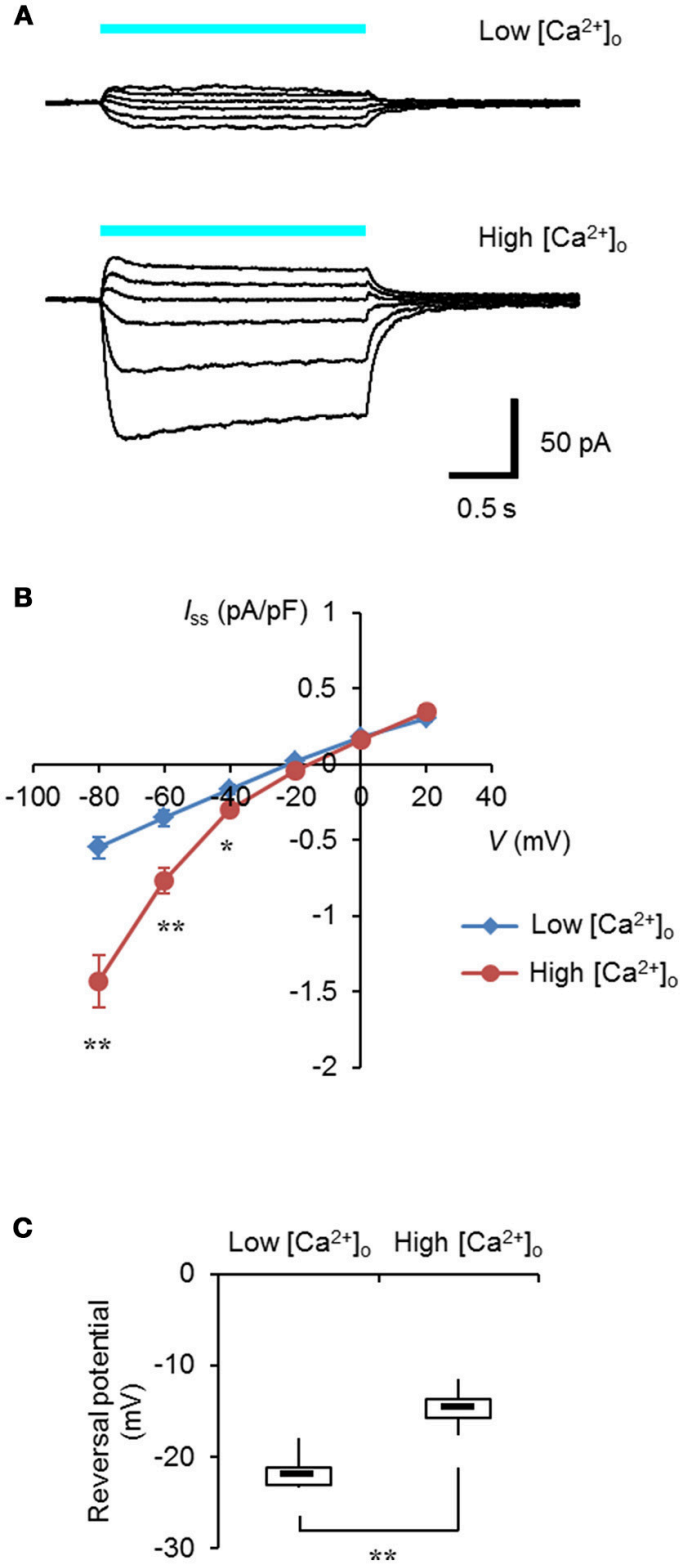
Ca^2+^-permeability of ChRGR. **(A)** Light-evoked whole cell currents from a ND 7/23 cell expressing ChRGR in a extracellular solution (pH 8.4) containing 0.1 mM [Ca^2+^]_o_ (Low, top traces; whole cell capacitance = 37 pF), or from another cell with 10 mM [Ca^2+^]_o_ (High, bottom traces; 39 pF), both at holding potentials of −80, −60, −40, −20, 0, and 20 mV. Each cyan line shows the timing of the irradiation (475 ± 28 nm, 1.3 mWmm^−2^). **(B)** The current-voltage (I-V) relationship of the steady-state photocurrent (*I*_ss_). Mean ± SEM (Low: *n* = 6, High: *n* = 7) after standardized by the cell capacitance and expressed as pA/pF. **(C)** Box-and-whisker plots comparing the reversal potential between Low (*n* = 6) and High (*n* = 7) [Ca^2+^]_o_. ^*^P < 0.05 and ^**^P < 0.005, Mann-Whitney *U*-test.

It was expected that selective control of the intracellular Ca^2+^ dynamics could be achieved by the specific targeting of light-sensitive actuators to the ER/SR membrane. Expanding the optogenetic toolbox enabled us to choose the optimal light-sensitive actuator depending on the experimental context (Mattis et al., 2011; Schneider et al., 2013). One of the chimeric channelrhodopsins, ChRGR, was characterized by the red-shifted light absorbance spectrum and lower desensitization over ChR2 (Wen et al., 2010), and it effectively facilitated myogenesis in a manner dependent on light when expressed in C2C12 myoblasts (Asano et al., 2015). As shown in Figures 1A,B, when ChRGR was expressed in ND 7/23 cells, which are optimal for the voltage control with negligible dye coupling (Hososhima et al., 2015), showed inward-rectifying photocurrents in the presence of 10 mM [Ca^2+^]_o_ and not with 0.1 mM [Ca^2+^]_o_. Indeed, the reversal potential of the photocurrent was −14 ± 0.8 mV in 10 mM [Ca^2+^]_o_ whereas it was −22 ± 0.8 mV in 0.1 mM [Ca^2+^]_o_ with significant difference (Figure 1C). Therefore, ChRGR would be suitable for optogenetic manipulation of the Ca^2+^ dynamics.

**Figure 2:**
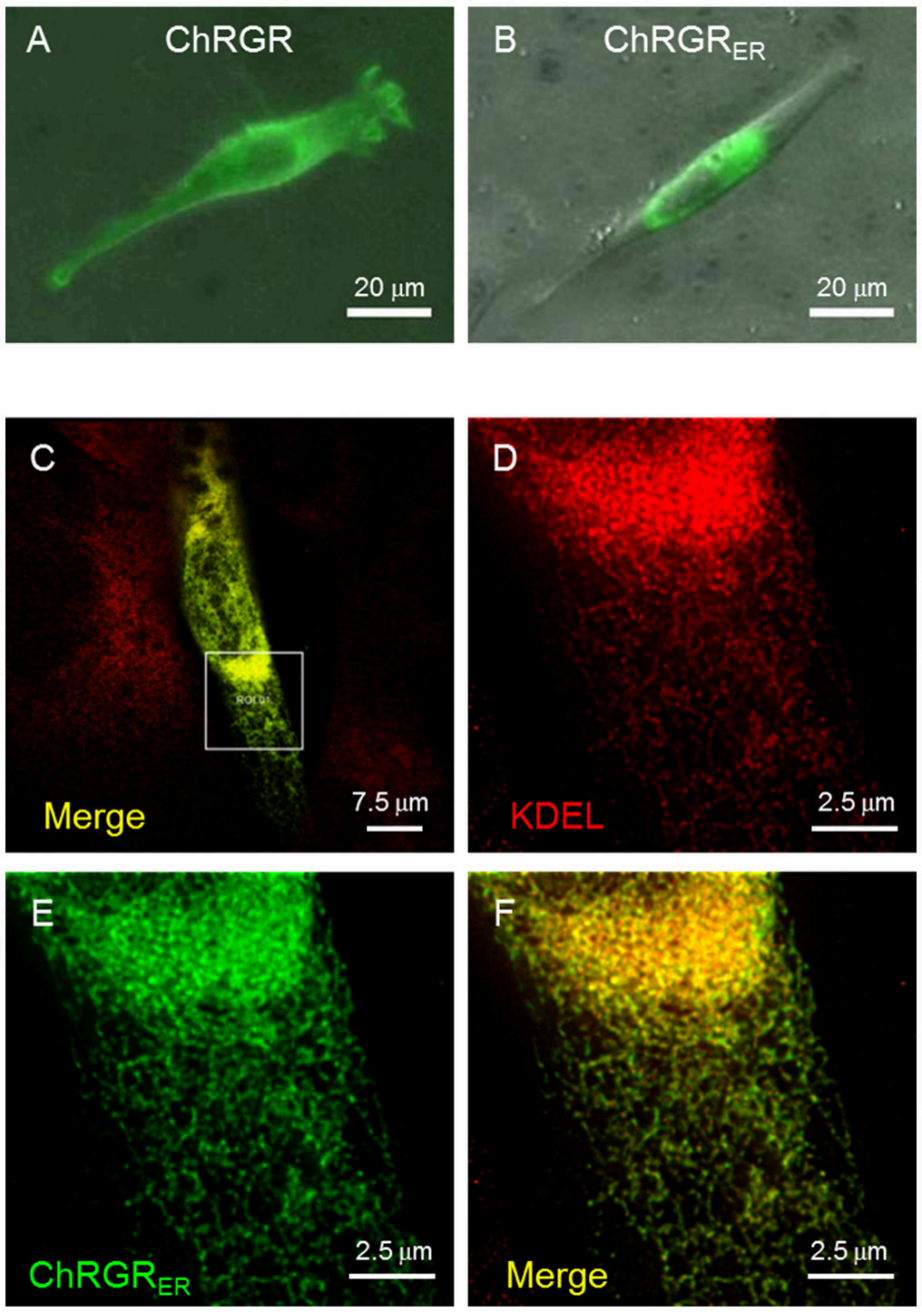
Cellular expression of ChRGR_ER_. **(A)** Image of Venus fluorescence of a typical C2C12 cell expressing ChRGR. **(B)** Another typical C2C12 cell expressing ChRGR_ER_. **(C)** Merged image of super-resolution microscopy (STED) of C2C12 immunostained for Venus (green) and ER-marker KDEL (red) at low magnification. **(D–F)** The ROI encircled in **(C)** was enlarged for each color channel; KDEL (**D**, red), Venus (**E**, green), and the merge **(F)**.

We then tested the ER/SR localization of ChRGR, connecting a sequence consisting of the 3rd-4th transmembrane helices of mouse ryanodine receptor 2 at its C-terminus end (hereafter called ChRGR_ER_). The expression patterns of ChRGR and the ChRGR_ER_ were visualized by Venus, which was tagged at their C-terminus (Figures 2A,B). The signal of ChRGR_ER_-Venus was confined in the peri-nucleus region in contrast to the conventional ChRGR-Venus, which localized in the plasma membrane with the original membrane targeting property (Wen et al., 2010; Asano et al., 2015). To further examine the precise localization, ChRGR_ER_-expressing C2C12 cells were immunostained for ER/SR-marker KDEL and subsequently observed under the STED super-resolution microscopy (Figures 2C–F). As shown in Figure 2F and Movie S1, the Venus fluorescence was mostly co-localized with the KDEL signal.

**Figure 3:**
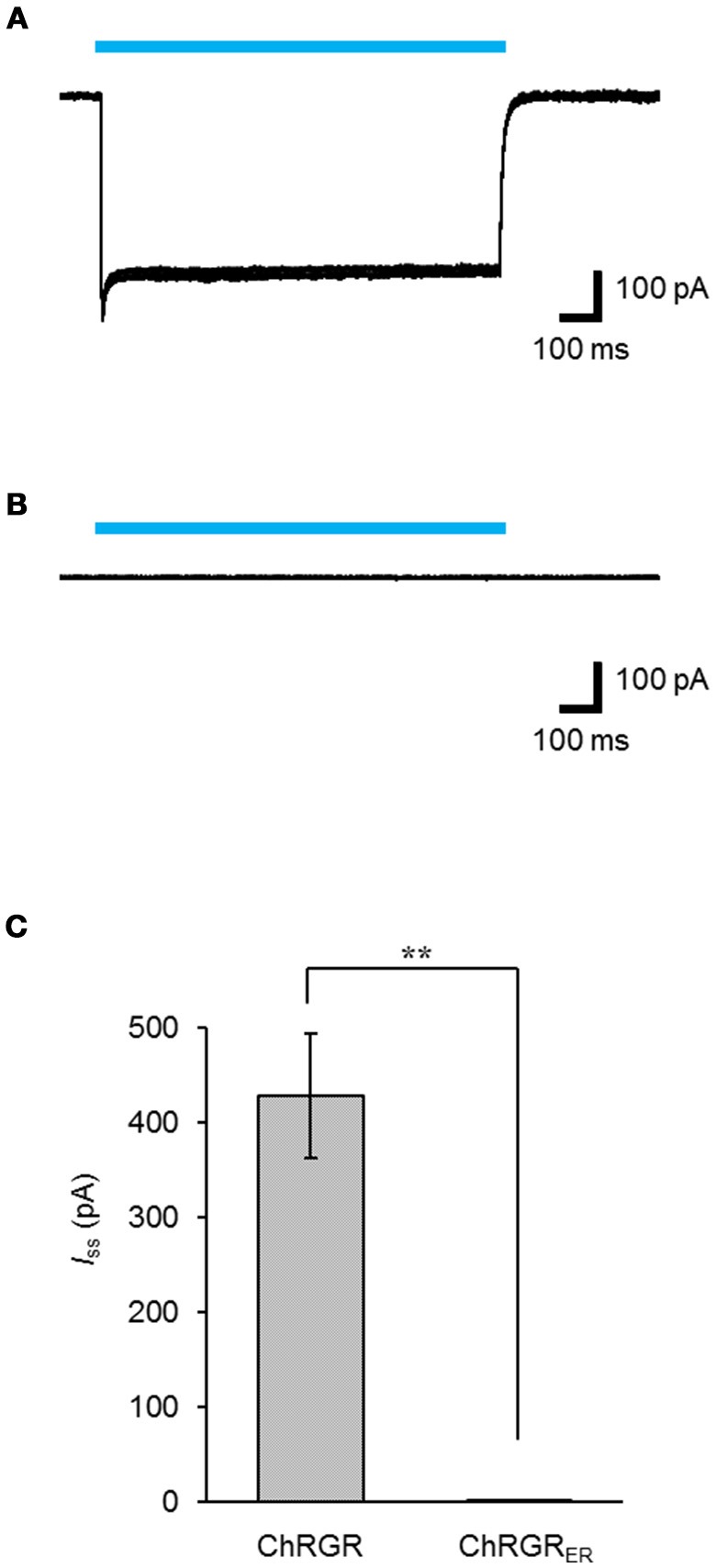
Intracellular distribution of ChRGR_ER_. **(A,B)** Overlay of sample photocurrents recorded from either a ChRGR-expressing C2C12 cell **(A)** or ChRGR_ER_-expressing one **(B)**. Each cyan line shows the timing of the LED irradiation (505 ± 15 nm, 1.6 mWmm^−2^). **(C)** Summary of the *I*_ss_ from the ChRGR- (*n* = 6) and ChRGR_ER_-expressing C2C12 cells (*n* = 7). ^**^p < 0.005, Mann-Whitney *U*-test.

Leaked expression of ChRGR_ER_ in the plasma membrane caused by miss-targeting or overexpression would hamper the precise regulation of the intracellular Ca^2+^ dynamics. To determine whether functional ChRGR_ER_ was incorporated in the plasma membrane, we performed patch clamp experiments under the whole-cell voltage clamp configuration. Cyan light (505 ± 15 nm; 1.58 mWmm^−2^; 1 s) evoked a photocurrent of 428 ± 65 pA (*n* = 6) in the ChRGR-expressing cells (Figure 3A). On the other hand, the photocurrent was negligible for the ChRGR_ER_-expressing cells (*n* = 7) even with the same light stimulation (Figure 3B), with significant difference (Figure 3C).

### Functional operation of ChRGR_ER_ in the cell

**Figure 4:**
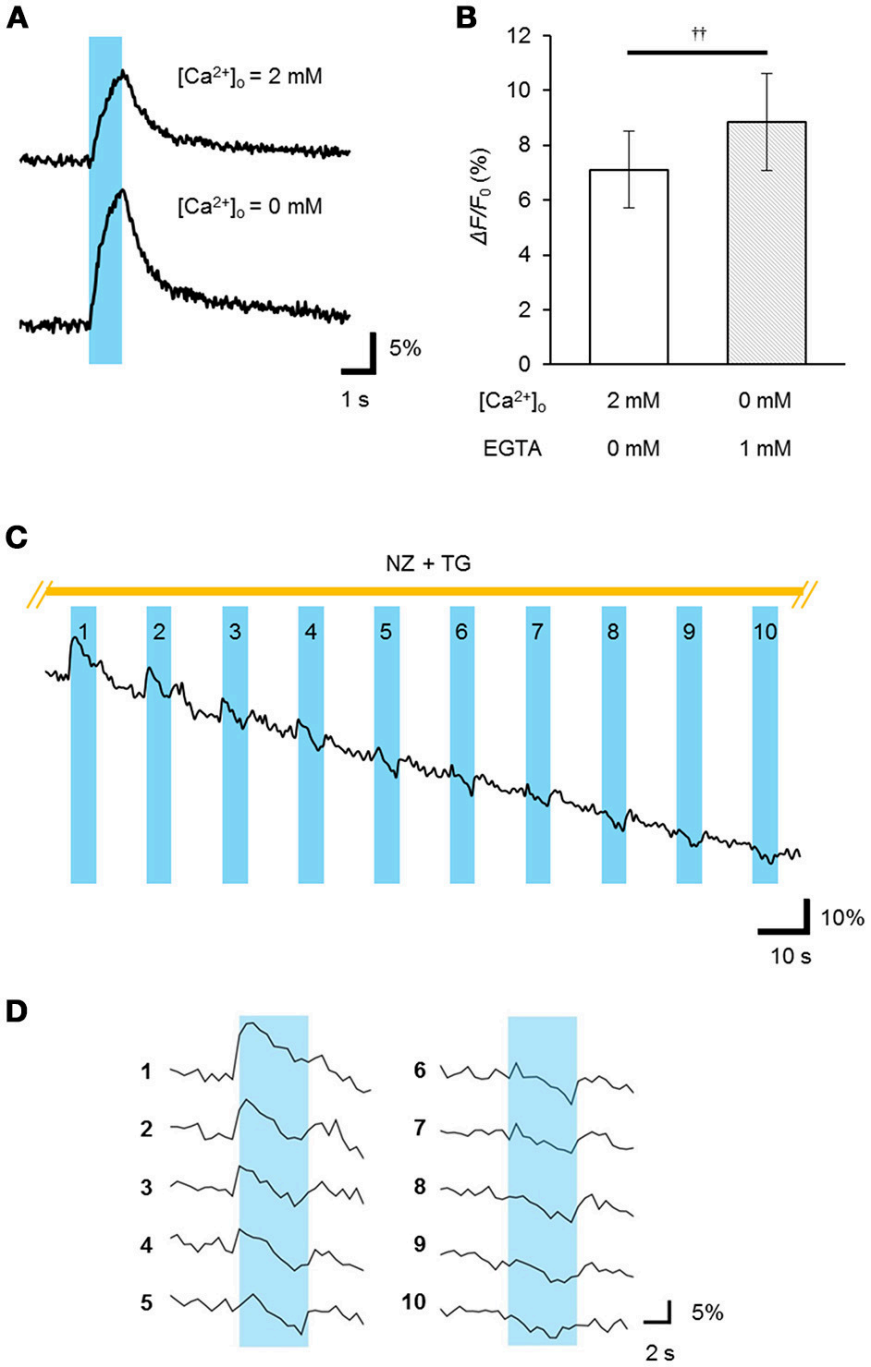
Light-evoked release of Ca^2+^ from the intracellular store. **(A)** The R-CaMP1.07 fluorescence increment (Δ*F*/*F*_0_) was recorded from a ChRGR_ER_-expressing C2C12 cell during optical stimulation (OS: a cyan stripe; 450 ± 10 nm, 5 ms pulse at 20 Hz for 1 s) applied in the presence (top) or absence (bottom) of [Ca^2+^]_o_. **(B)** Summary of the peak Δ*F*/*F*_0_ (*n* = 7).*p* < 0.005, Wilcoxon signed rank test. **(C)** The light-dependent store depletion. The R-GECO1 signal (Δ*F*/*F*_0_) was sampled at 2 Hz while the OS (cyan stripes; 451 nm; duration, 20 ms; 10 Hz for 5 s) was repetitively applied at every 15 s to a ChRGR_ER_-expressing C2C12 cell in a [Ca^2+^]_o_-free extracellular solution containing EGTA (5 mM), nifedipine (NZ, 10 μM) and thapsigargin (TG, 2 μM). **(D)** The Δ*F*/*F*_0_ signal was extracted from **(C)** for each cycle of OS. Each number indicate the order of the cycles.

To determine whether the ChRGR_ER_ was fully functional in the ER/SR, we performed real-time Ca^2+^ imaging using the red fluorescent Ca^2+^ indicator R-CaMP1.07 (Ohkura et al., 2012). The double positive cells with ChRGR_ER_-Venus and R-CaMP1.07 were identified and stimulated with a train of pulses (5 ms at 20 Hz for 1 s) of blue LED light (4.5 mWmm^−2^ at 450 nm). In the standard extracellular milieu containing 2 mM Ca^2+^, the ChRGR_ER_-expressing cells responded to the light by 7.1 ± 1.4% (*n* = 11) red fluorescence Δ*F*/*F*_0_ (Figures 4A,B). When the perfusing solution was switched to the [Ca^2+^]_o_-free solution containing 1 mM EGTA, the Δ*F*/*F*_0_ signal was significantly enhanced to 8.8 ± 1.8% by the same light. However, the light-dependent Δ*F*/*F*_0_ was reduced with the repetitive OS when the Ca^2+^ entry was suppressed by the external EGTA and nifedipine and the ER/SR uptake were blocked by thapsigargin (Figures 4C,D). In fact, the Δ*F*/*F*_0_ ranged from −15 to −11% of the initial value at the end of the repetition (10–50 cycles, *n* = 3).

**Figure 5:**
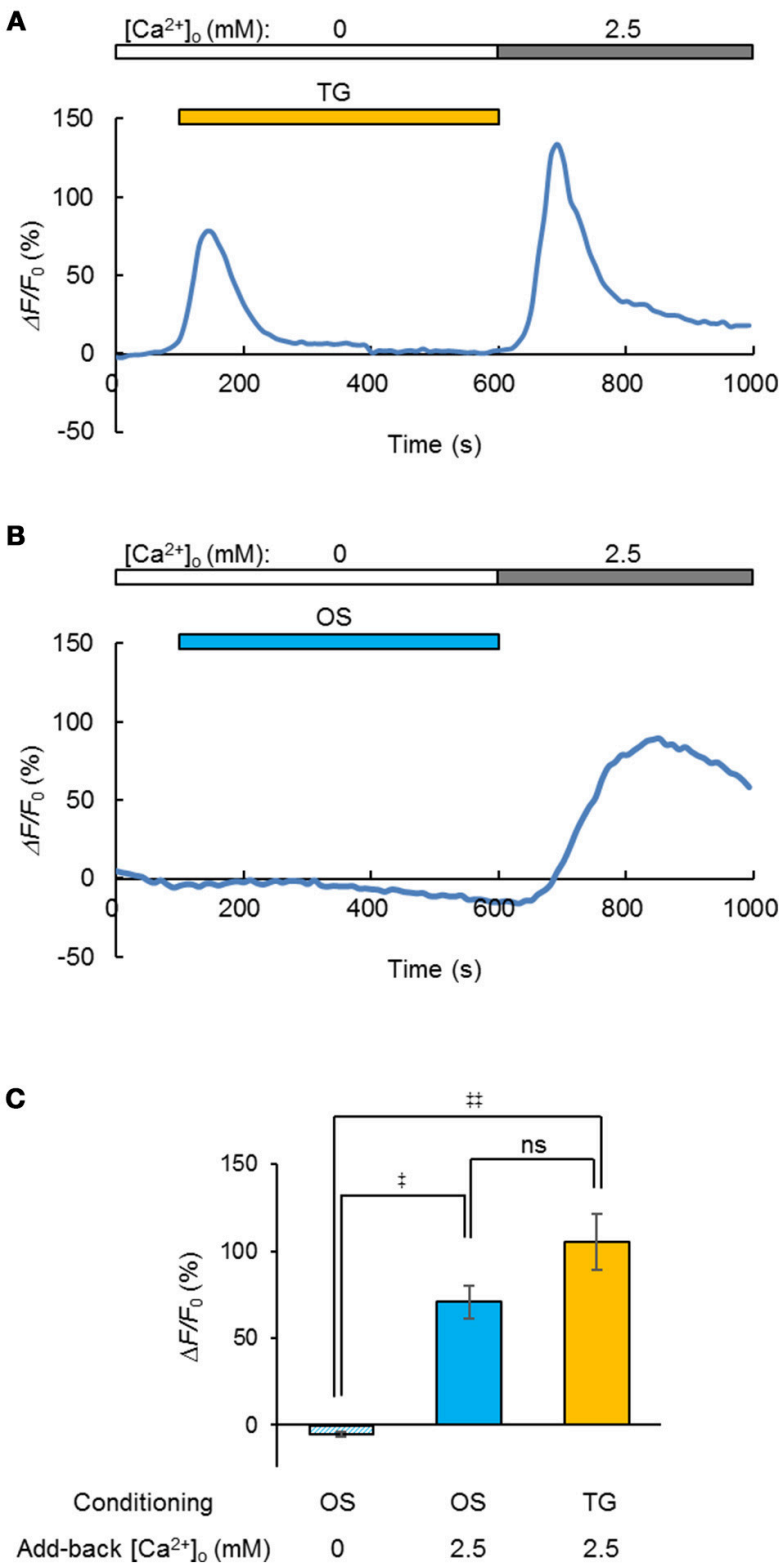
Light-induced store-operated Ca^2+^ entry (SOCE). **(A)** The SOCE was observed by the [Ca^2+^]_o_ add-back from 0 to 2.5 mM after depletion of the intracellular Ca^2+^ store by thapsigargin (TG, orange stripe, 5 μM), while the change of R-GECO1 fluorescence (Δ*F*/*F*_0_) was recorded (0.1 fps) from a ChRGR_ER_-expressing C2C12 cell. **(B)** Similar to **(A)**, but the OS (blue stripe, 475 ± 10 nm, 10 ms pulse at 20 Hz for 0.5 s) was repetitively applied (50 cycles at every 10 s). Note that the OS increased the fluorescence only slightly because it was applied between the sampling of images. **(C)** Summary of the OS-induced SOCE: without add-back [Ca^2+^]_o_ after OS (left, *n* = 7), with add-back [Ca^2+^]_o_ after OS (*n* = 12) and with add-back [Ca^2+^]_o_ after TG (*n* = 8).^‡^*P* < 0.05; ^‡‡^*P* < 0.01; ns, *P* > 0.1; one-way Kruskal-Wallis test by ranks.

Generally, the depletion of Ca^2+^ from the ER/SR induced Ca^2+^ entry through the plasma membrane and facilitated the subsequent uptake of Ca^2+^ (store-operated Ca^2+^ entry, SOCE) by the coupling between STIM and Orai proteins (Prakriya and Lewis, 2015). Indeed, when the Ca^2+^ store in a C2C12 cell was depleted by treatment with thapsigargin (5 μM) in a [Ca^2+^]_o_-free milieu, the entry of Ca^2+^ was evident from the [Ca^2+^]_i_ increase upon the add-back of [Ca^2+^]_o_ to 2.5 mM (Figure 5A). Similarly, a significant Ca^2+^ entry was observed when the OS was repetitively applied before the [Ca^2+^]_o_ add-back (Figure 5B), yet was negative in a [Ca^2+^]_o_-free milieu (Figure 5C). In summary, a significant SOCE was observed after OS of the ChRGR_ER_-expressing C2C12 cells (Figure 5C).

## Discussion

**Figure 6:**
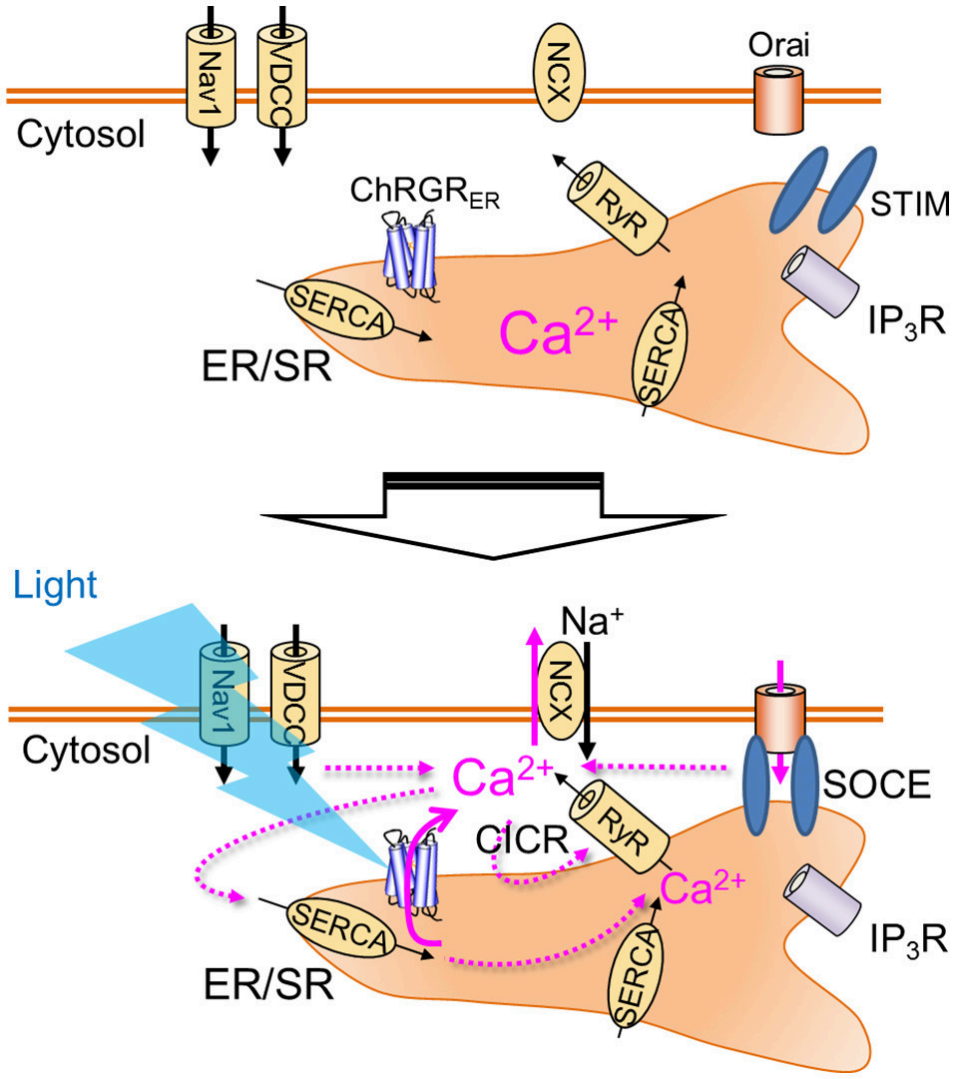
Organelle optogenetics. In the present study, the endoplasmic reticulum/sarcoplasmic reticulum (ER/SR)-targeted channelrhodopsin, ChRGR_ER_ was selectively expressed in the ER/SR membrane. The light absorption by this molecule triggers release of Ca^2+^ from the intracellular store, which activates various Ca^2+^ signaling cascades such as calcium-induced calcium release (CICR) or store-operated calcium entry (SOCE). Nav1, voltage-dependent sodium channel 1; VDCC, voltage-dependent calcium channel; NCX, Na^+^-Ca^2+^ exchanger; RyR, ryanodine receptor; IP_3_R, IP_3_ receptor; SERCA, sarco/endoplasmic reticulum Ca^2+^-ATPase.

This paper demonstrated for the first time specific control of intracellular Ca^2+^ dynamics by light with the application of organelle optogenetics using an ER/SR-targeted channelrhodopsin, ChRGR_ER_, that includes the 4th transmembrane helix of ryanodine receptor as an ER-retention motif (Bhat and Jianjie, 2002). This conclusion was supported by the following evidences: (1) the localization pattern of ChRGR_ER_ was 3-dimensionally merged with the ER markers even when examined under the super-resolotion microscopy; (2) whole cell patch clamp recording detected the photocurrent in the ChRGR-expressing C2C12 cells but not in the ChRGR_ER_-expressing ones; (3) the fluorometric Ca^2+^ signal (Δ*F*/*F*_0_) was induced by the OS in the ChRGR_ER_-expressing cells even in the absence of [Ca^2+^]_o_ (see also Movie S2). It was even larger than that in the presence of [Ca^2+^]_o_, probably due to the reduction of basal [Ca^2+^]_i_; (4) the Δ*F*/*F*_0_ was sensitive to the SERCA inhibitor (thapsigargin). (5) the SOCE was induced by the OS in the ChRGR_ER_-expressing cells. The organelle optogenetics (Figure 6) would thus enable the regulation of not only the intracellular Ca^2+^ release from ER/SR but also the subsequent activation of other internal Ca^2+^ signaling cascades such as calcium-induced calcium release (CICR) (Endo et al., 1977) or SOCE (Soboloff et al., 2012; Bagur and Hajnóczky, 2017). Although prevalent in every cell, tissue, and organ of any living organism, the consequences of the spatiotemporal dynamics of intracellular Ca^2+^ have not been extensively studied because of technical limitations in differentiating between the two major sources of Ca^2+^ mobilization: the extracellular milieu and the internal Ca^2+^ stores. Therefore, the present optogenetic manipulation of ER/SR would represent a breakthrough in elaborating its physiological significance, such as the triggering of the sarcomere assembly (Ferrari et al., 1996, 1998; Li et al., 2004). Accumulating evidence has indicated that intracellular Ca^2+^ stores similarly have an important functional role in neurons; for example, somatodendritic signaling, synaptic transmission and plasticity (Simpson et al., 1995; Bouchard et al., 2003; Collin et al., 2005; Segal and Korkotian, 2014, 2015) in addition to being involved in neurodegenerative diseases such as Alzheimer's (Villegas et al., 2014; Zhang et al., 2016). Further improvements in optogenetic molecular tools, targeting and expression techniques, and optical systems will enable the precise manipulation of intracellular Ca^2+^ in neuroscience.

## Author contributions

TA, HI, TI, and HY conceived, designed, and performed the experiments, and analyzed the data. HI, TI, and HY contributed reagents, materials, and analysis tools. All authors drafted and reviewed the manuscript.

### Conflict of interest statement

The authors declare that the research was conducted in the absence of any commercial or financial relationships that could be construed as a potential conflict of interest.

## Supplementary material

Movie S1 Three-dimensional expression of ChRGR_ER_ in a cell. Images of super-resolution microscopy (STED) of C2C12 immunostained for Venus (green) and ER-marker KDEL (red) were merged for the same ROI shown in Figure 1C. Scale, 2 μm.

Movie S2 Representative movies of light-induced intracellular Ca^2+^ increase in a ChRGR_ER_-expressing C2C12 cell. **(A,B)** The R-GECO1 signals were sampled at 2 Hz and the fluorescence intensity (*F*) was imaged during an optical stimulation (OS: 451 nm; duration, 20 ms; 10 Hz for 5 s) in the presence **(A)** and absence **(B)** of extracellular Ca^2+^ ([Ca^2+^]_o_). The images were sampled at 2 Hz and pseudocolor-displayed at 20 Hz. Scale bar, 10 μm. **(C,D)** The changes (Δ*F/F*_0_) of R-GECO1 fluorescence of ROIs above the cells shown in **(A,B)**, respectively. Each OS was indicated as a cyan stripe.
