# Supplementary material for: Organelle Optogenetics: Direct Manipulation of Intracellular Ca2+ Dynamics by Light
Source: Front Neurosci. 2018 Aug 17;12:561. doi: 10.3389/fnins.2018.00561 (PMC6107701; doi:10.3389/fnins.2018.00561)
Supplement: Movie S2 — Representative movies of light-induced intracellular Ca2+ increase in a ChRGRER-expressing C2C12 cell. (A,B) The R-GECO1 signals were sampled at 2 Hz and the fluorescence intensity (F) was imaged during an optical stimulation (OS: 451 nm; duration, 20 ms; 10 Hz for 5 s) in the presence (A) and absence (B) of extracellular Ca2+ ([Ca2+]o). The images were sampled at 2 Hz and pseudocolor-displayed at 20 Hz. Scale bar, 10 μm. (C,D) The changes (ΔF/F0) of R-GECO1 fluorescence of ROIs above the cells shown in (A,B), respectively. Each OS was indicated as a cyan stripe. [file Presentation_1.pptx]

## Slide 1
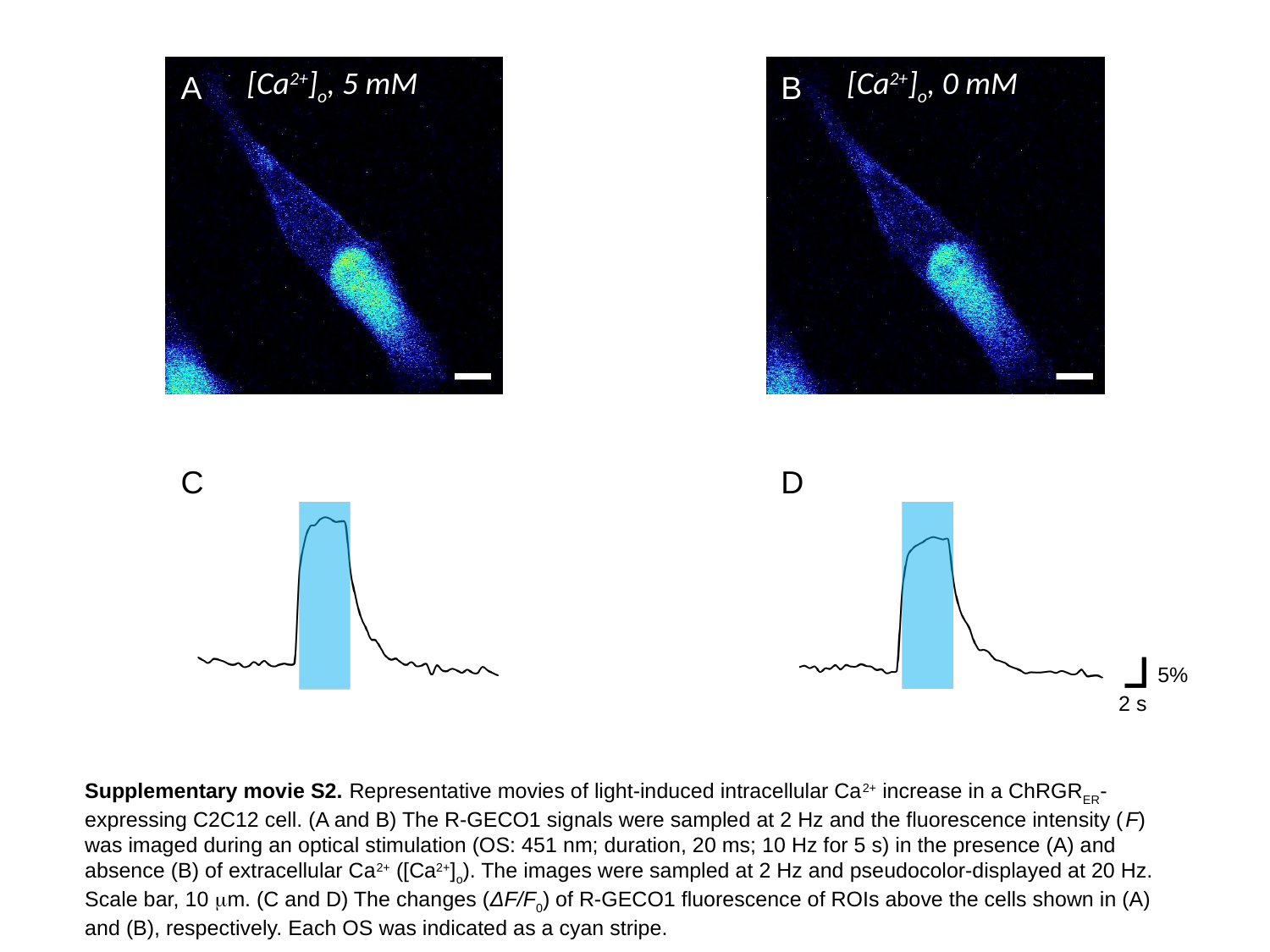

A
B
[Ca2+]o, 5 mM
[Ca2+]o, 0 mM
C
D
5%
2 s
Supplementary movie S2. Representative movies of light-induced intracellular Ca2+ increase in a ChRGRER-expressing C2C12 cell. (A and B) The R-GECO1 signals were sampled at 2 Hz and the fluorescence intensity (F) was imaged during an optical stimulation (OS: 451 nm; duration, 20 ms; 10 Hz for 5 s) in the presence (A) and absence (B) of extracellular Ca2+ ([Ca2+]o). The images were sampled at 2 Hz and pseudocolor-displayed at 20 Hz. Scale bar, 10 mm. (C and D) The changes (ΔF/F0) of R-GECO1 fluorescence of ROIs above the cells shown in (A) and (B), respectively. Each OS was indicated as a cyan stripe.
